# Supplementary material for: Nasal bots carry relevant titers of CWD prions in naturally infected white-tailed deer
Source: EMBO Rep. 2024 Jan 8;25(1):334–50. doi: 10.1038/s44319-023-00003-7 (PMC10883265; doi:10.1038/s44319-023-00003-7)
Supplement: Supplementary file 1 — Appendix [file 44319_2023_3_MOESM1_ESM.pdf]

Appendix to:

Nasal bots carry relevant titers of CWD prions in naturally infected white-tailed deer.

TABLE OF CONTENTS

Appendix Table S1 ………………………………………………………………………….. Page 2

Appendix Figure S1 ………………………………………………………………………….. Page 3

Appendix Figure S2 ………………………………………………………………………….. Page 4

Appendix Figure S3 ………………………………………………………………………….. Page 5

Appendix Figure S4 ………………………………………………………………………….. Page 6

Appendix Figure S5 ………………………………………………………………………….. Page 7

Appendix Figure S6 ………………………………………………………………………….. Page 8

Appendix Figure S7 ………………………………………………………………………….. Page 9

Appendix Figure S8 ………………………………………………………………………….. Page 11

<u>Appendix Table S1.</u> Detection of CWD-prions in nasal bots collected from deer in areas with different CWD prevalence.

**Location**	**CWD on site**	**Free-ranging or Farmed**	**Positive Nasal bot**	**Negative Nasal bot**
TX-1	Yes (High prevalence)	Farmed	8	0
MW-1	No (Low prevalence)	Farmed	0	9
MW-2	Yes (High prevalence)	Farmed	4	0
Del Rio, Texas	Yes (Low prevalence)	Free-ranging	0	62
Texas	Unknown	Farmed	6	1
Other sites	Yes (High prevalence)	Farmed	3	3

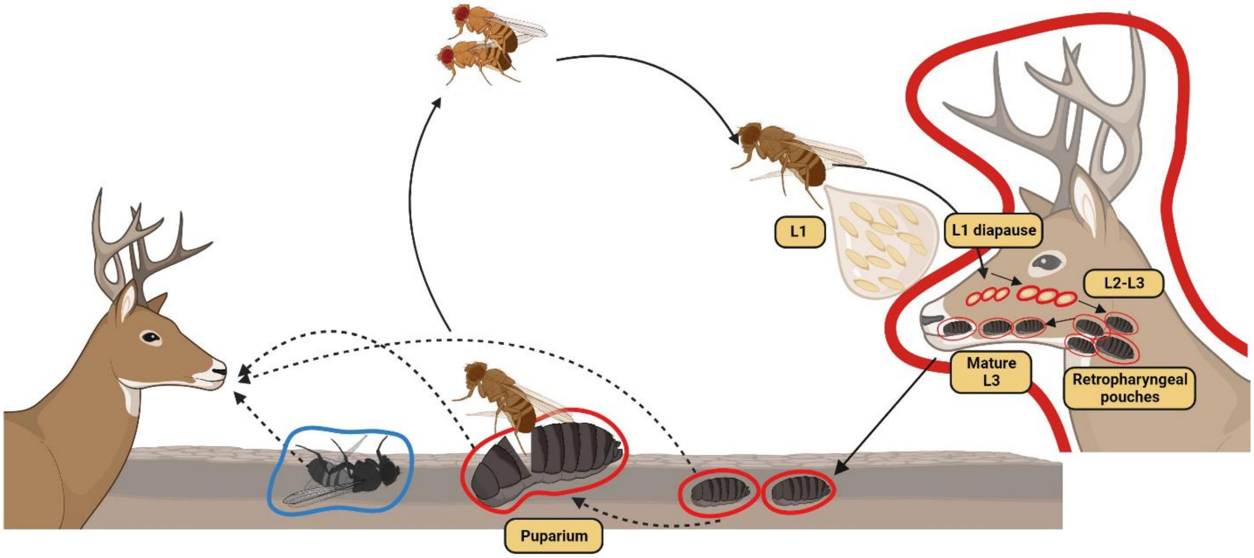

<u>Appendix Figure S1.</u> Schematic representation of the life cycle of nasal bots, their interaction with deer, and their potential role as CWD vectors. L1, L2 and L3 represent different larva stages. Structures circled in red indicate CWD prion contaminated organisms. The fly circled in blue represent the hypothetical scenario in which a mature bot fly contain disease relevant CWD prions. Dotted lines depict the potential interaction between CWD infected parasites and naïve deer. Scheme created by BioRender.

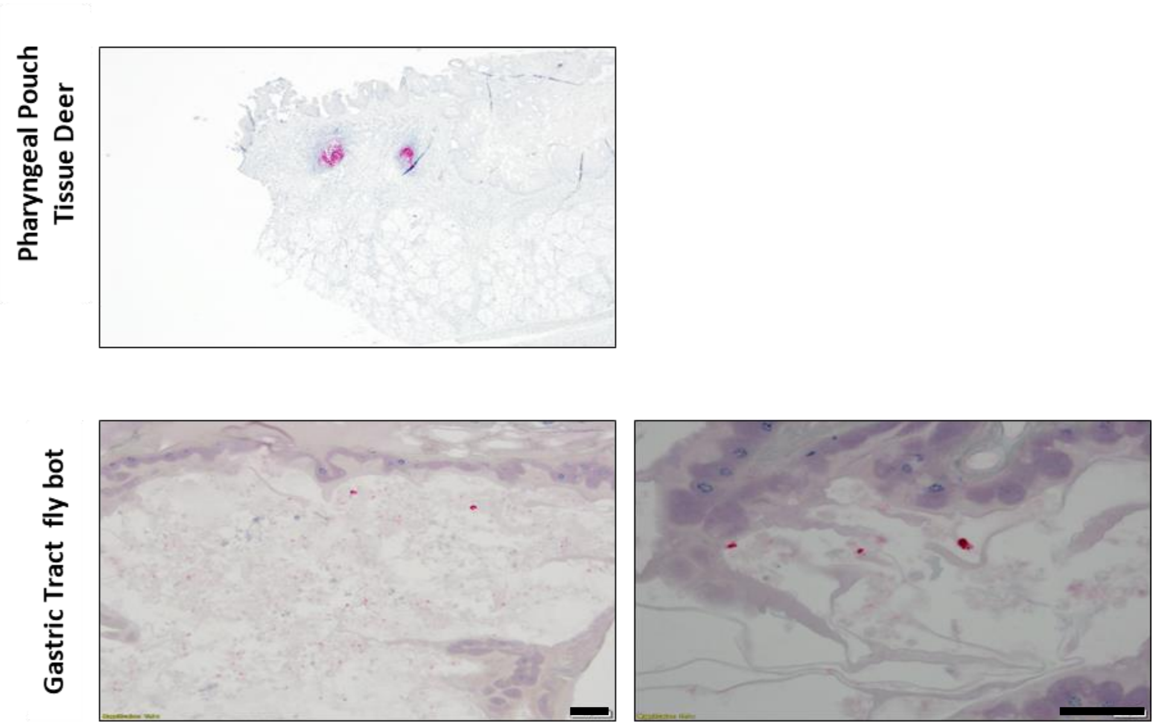

<u>Appendix Figure S2.</u> **Immunohistochemical analysis of nasal bots collected from a CWD-infected white-tailed deer. A)** Pharyngeal pouch tissue of a non-clinical, CWD-infected white-tailed deer. Red inclusions depict accumulation of PK-resistant prion protein. **B) and C)** Micrographs depicting prion inclusion (red dots) in the gastro-intestinal system of nasal bots collected from a CWD-infected white-tailed deer. Bar in B) represents 50 µm and bar in C) represents 20 µm.

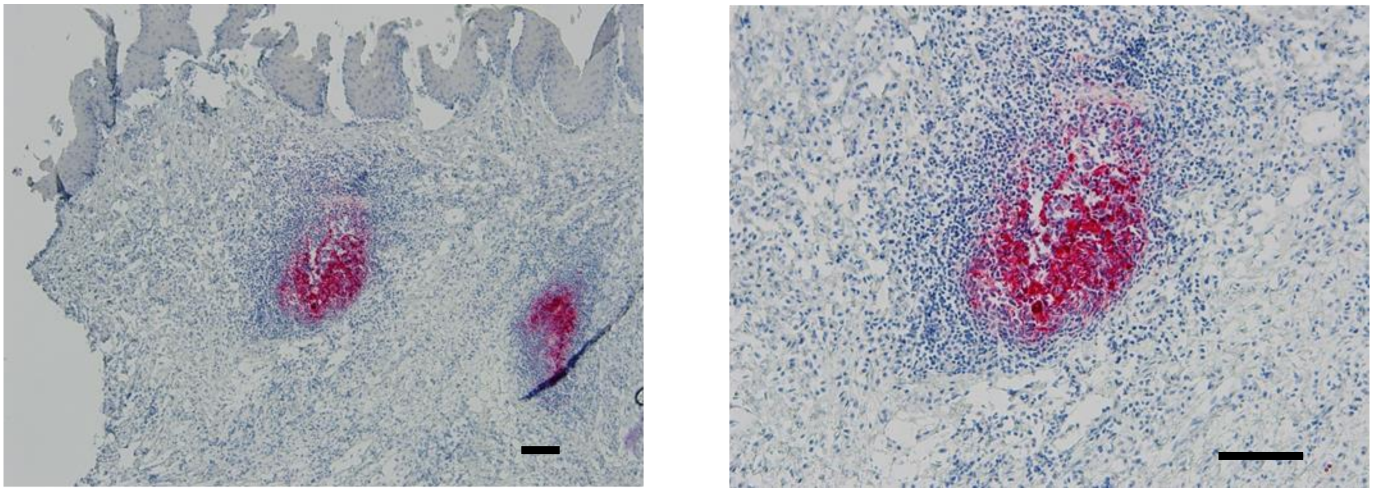

<u>Appendix Figure S3.</u> Higher magnifications of PrP^{Sc} staining in pharyngeal pouches of a CWD-infected, non-clinical white-tailed deer. Images were taken at 10X (left) and 20X (right) magnification. Bars represent 10 (left panel) µm and 20 µm (right panel).

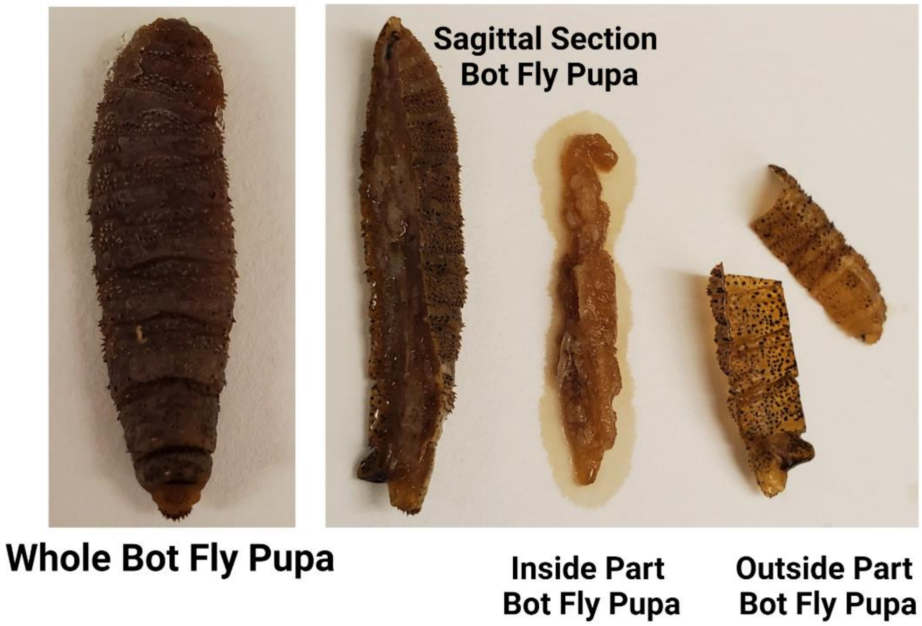

Appendix Figure S4. **Representative picture of bot flies and bot flies' components (protective shells and inside components) used in this study.**

Mouse Bioassay

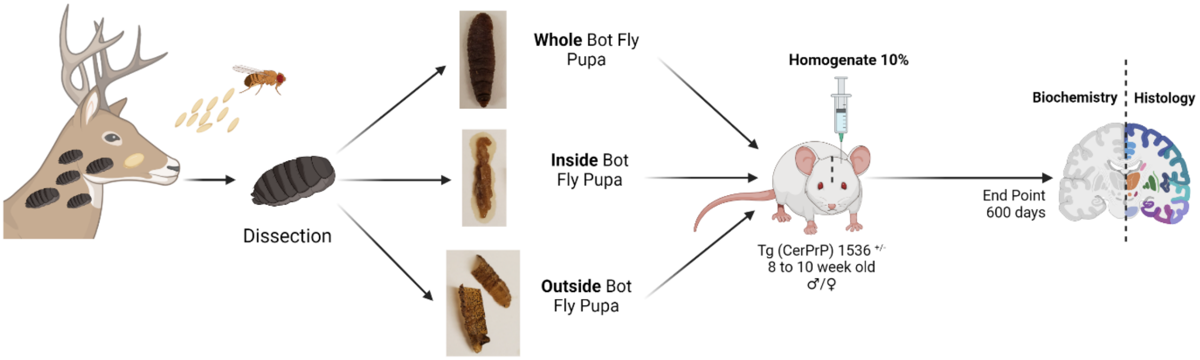

<u>Appendix Figure S5.</u> Experimental strategy used for bioassays. Homogenates from bots collected from captive white-tailed deer were intra-cerebrally inoculated in the brains of tg1536$^{+/-}$ mice. Mice were sacrificed when showing prion-associated clinical signs or after 600 days. Brains collected from these mice were divided into their both hemispheres and stored for biochemical and immunohistochemical analyses as explained in Methods.

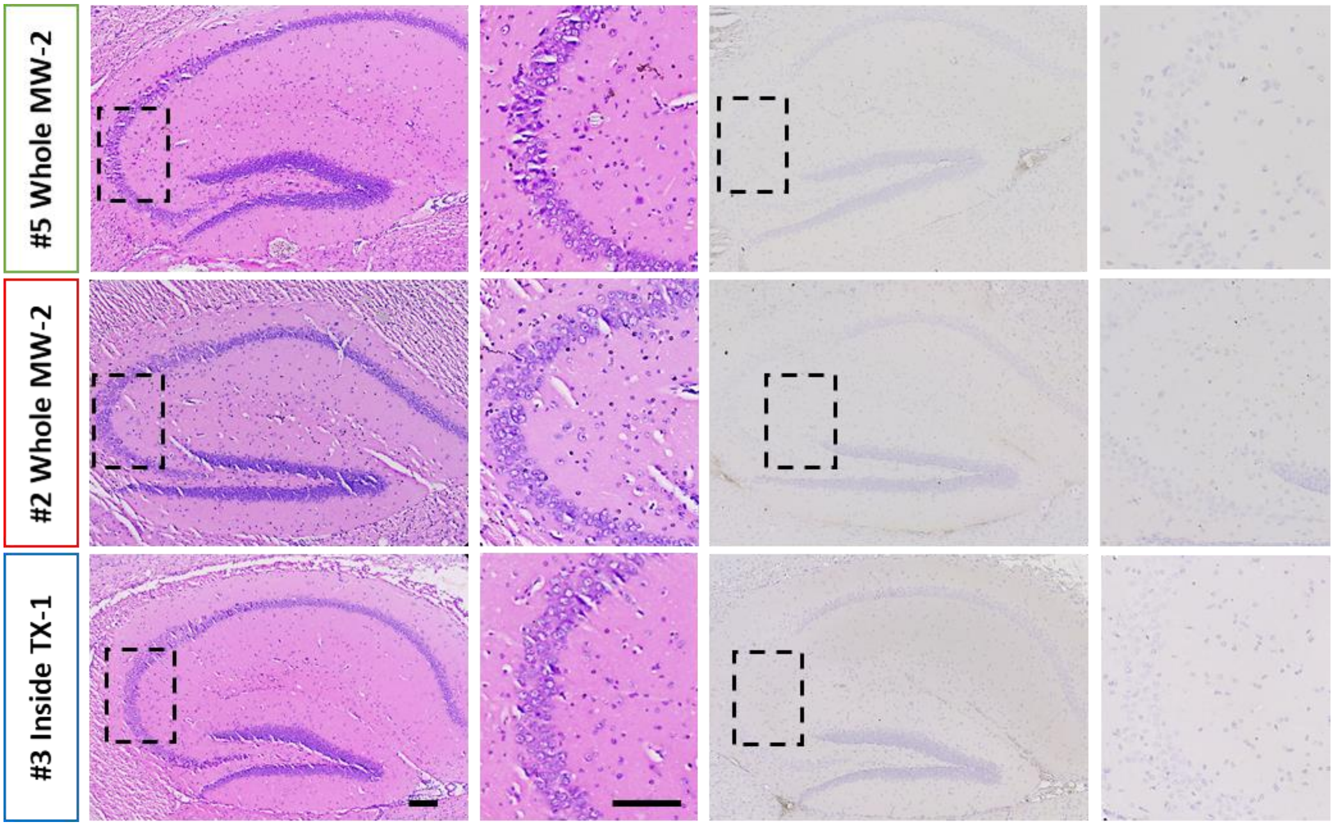

<u>Appendix Figure S6.</u> Histological characteristics of mice's brains diagnosed as CWD-negative after western blot and PMCA analyses. Representative brain images of mice determined as CWD-negative after western blot and PMCA analyses. Dotted squares represent areas of interest that were magnified and shown at the right of each panel. Bars represent 100 µm.

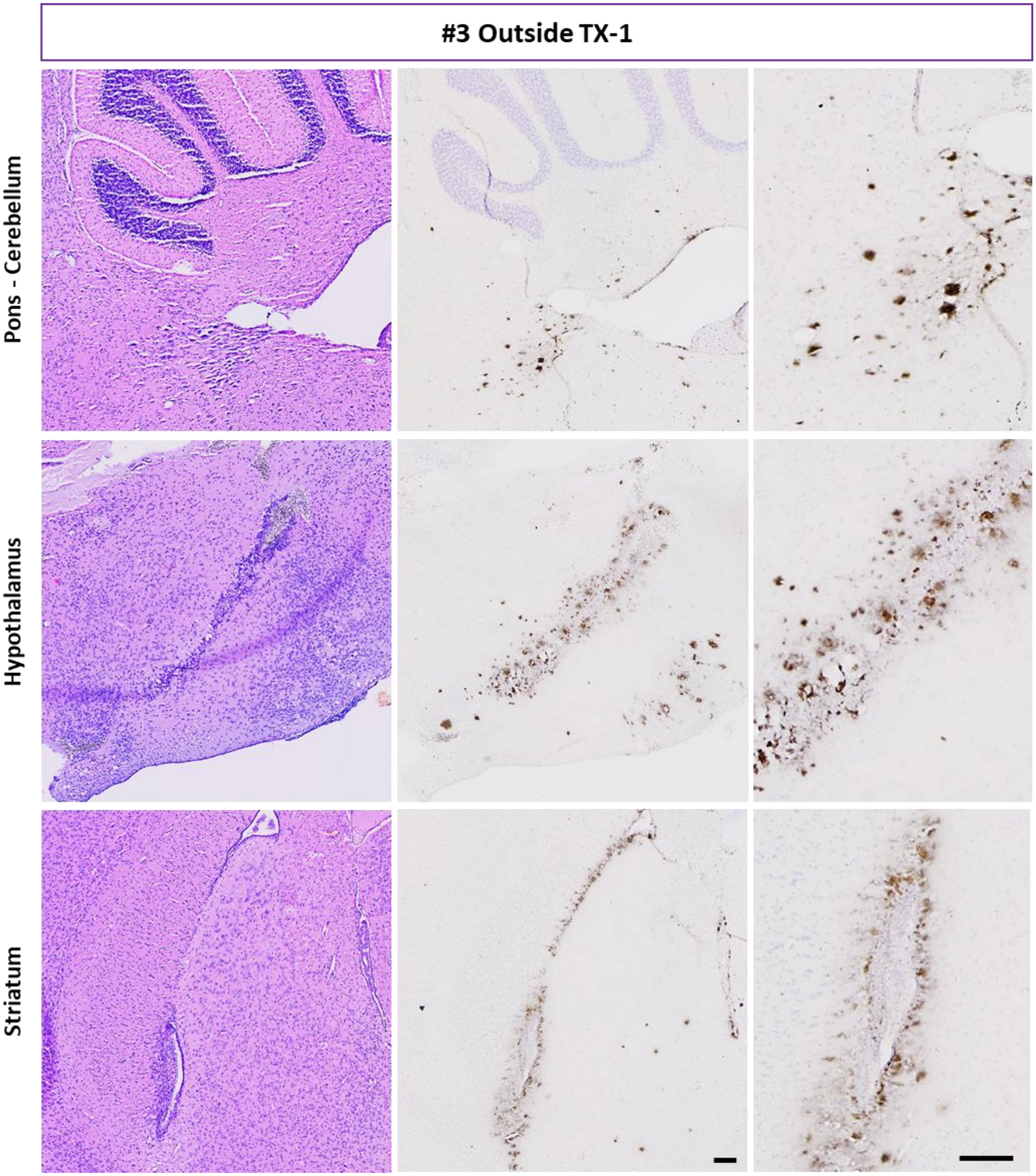

<u>Appendix Figure S7.</u> Overview of PrP^{Sc} deposition in mice's brains designated as CWD positive. Representative images of pons-cerebellum (top panels), hypothalamus (middle panels)

and striatum (lower panels) in mice determined as CWD-positive after western blot and PMCA analyses. Images at the left were stained with hematoxylin-eosin. Panels in the middle and right were probed for PrP^{Sc} as explained in Methods. Panels at the left are magnifications of panels in the middle. Bars represent 100 µm.

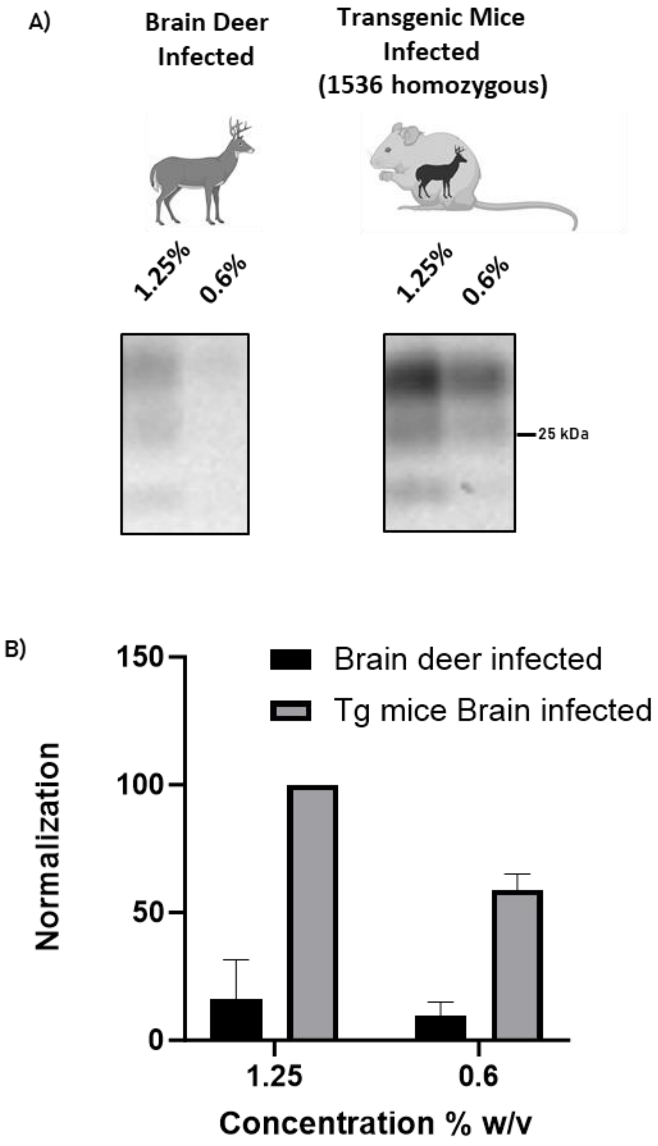

<u>Appendix Figure S8.</u> Comparison of the PrP^{Sc} levels in a terminally ill CWD-infected white-tailed deer and tg1536 mice. A) Western blots showing the levels of PrP^{Sc} in a terminally ill, CWD-infected white-tailed deer (left) and tg1536 mice (right). Two different brain dilutions (1.25% and 0.6% w/v) were tested in this experiment. The number at the right of the panel represent a molecular weight marker. **B)** Graph depicting normalized densitometric values of three independent experiments. Data is expressed as averages ± standard errors.
